# Efficient rare earth cerium(III) complex with nanosecond *d*−*f* emission for blue organic light-emitting diodes

**DOI:** 10.1093/nsr/nwaa193

**Published:** 2020-08-28

**Authors:** Zifeng Zhao, Liding Wang, Ge Zhan, Zhiwei Liu, Zuqiang Bian, Chunhui Huang

**Keywords:** cerium complex, doublet emission, *d−f* transition, organic light-emitting diodes

## Abstract

In the field of RGB diodes, development of a blue organic light-emitting diode (OLED) is a challenge because of the lack of an emitter which simultaneously has a short excited state lifetime and a high theoretical external quantum efficiency (EQE). We demonstrate herein a blue emissive rare earth cerium(III) complex Ce-2 showing a high photoluminescence quantum yield of 95% and a short excited state lifetime of 52.0 ns in doped film, which is considerably faster than that achieved in typical efficient phosphorescence or thermally activated delayed fluorescence emitters (typical lifetimes >1 μs). The corresponding OLED shows a maximum EQE up to 20.8% and a still high EQE of 18.2% at 1000 cd m^−2^, as well as an operation lifetime 70 times longer than that of a classic phosphorescence OLED. The excellent performance indicates that cerium(III) complex could be a candidate for efficient and stable blue OLEDs because of its spin- and parity-allowed *d−f* transition from the Ce^3+^ ion.

## Teaser

Unlike traditional emitters in organic light-emitting diodes (OLEDs), lanthanide cerium(III) complexes with d–f transition show simultaneously advantages of theoretical high efficiency, short excited state lifetime, adjustable emission color, and low cost.

## INTRODUCTION

During decades of efforts, theoretical 100% internal quantum efficiency (IQE) of organic light-emitting diodes (OLEDs) has been achieved using phosphorescence [1–4], thermally activated delayed fluorescence (TADF) [5–7] and organic radical [8,9] materials as emitters. At the same time, tremendous progress has been made in device operation lifetime, which has allowed for commercialization of high efficiency red and green OLEDs in display and lighting applications [10]. However, development of a blue OLED that combines high efficiency and long device operation lifetime, remains a challenge. In attempts to develop efficient blue phosphorescence and TADF emitters in OLEDs, the high-energy (>2.6 eV) and long excited state lifetime (around microseconds) triplet excitons can easily induce annihilation and/or chemical reactions at high current density, leading to efficiency roll-off and device degradation [11]. Therefore, much effort has been made in molecule design to shorten the excited state lifetime for better device stability [11,12]. Theoretically, the rare earth cerium(III) complex has a short excited state lifetime [13–16], and a high theoretical IQE up to 100%, ascribed to the spin- and parity-allowed doublet *d−f* transition of Ce^3+^ ions, although this concept has not been formally proposed and demonstrated. The emission wavelength of the cerium(III) complex could be adjusted by varying the coordinate environment [17], and the cost of cerium is much lower than that of iridium and platinum because of the rich abundance of cerium in earth (even higher than copper) and the simple isolation process from other lanthanide elements [18]. All these advantages reveal the huge potential of the cerium(III) complex in OLEDs. However, electroluminescence investigations on cerium(III) complexes are scarce and the reported maximum external efficiency (EQE) is <1% [19–21] because most reported cerium(III) complexes are non-emissive [22]. As a breakthrough, we demonstrate herein that cerium(III) complex Ce-2 shows a maximum EQE up to 20.8%, corresponding to an IQE close to 100%, and an operation lifetime (LT_70_) about 70 times longer than that of bis(4,6-difluorophenylpyridine)(picolinate)iridium (FIrpic) in OLEDs, arising from its doublet *d*−*f* transition mechanism and short excited state lifetime.

## RESULTS AND DISCUSSION

**Figure 1.**
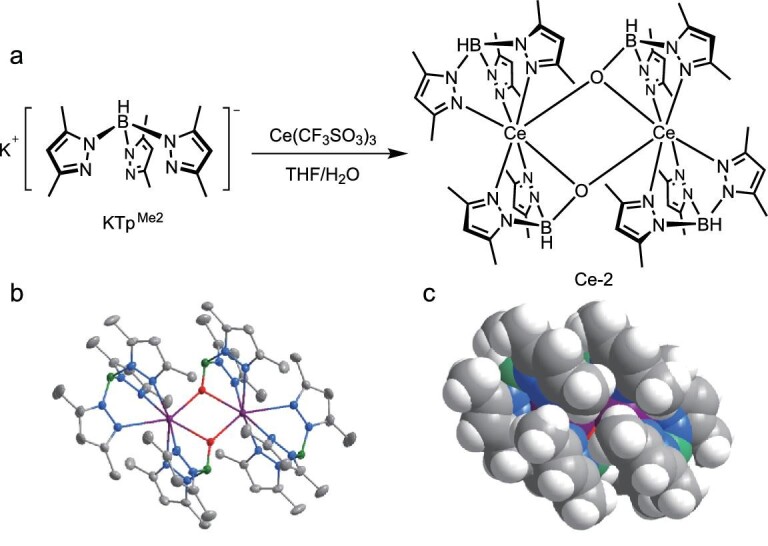
Synthesis and molecule structure of Ce-2. (a) Synthesis route of Ce-2. (b) Oak ridge thermal ellipsoid plot (ORTEP) drawing of Ce-2 at a 50% probability level, where purple is used for Ce, green for B, blue for N, red for O and gray for C. All hydrogen atoms have been omitted for clarity. (c) The space-filling view of Ce-2, where white is used for H.

The complex Ce-2 is synthesized by stirring potassium hydrotris(3,5-dimethylpyrazolyl)borate [23] (KTp^Me2^) with Ce(CF_3_SO_3_)_3_ in tetrahydrofuran, accompanied by hydrolysis from the presence of water (Fig. 1a). Although this reaction was discovered by accident, it is reproducible following the synthetic method showed in the Methods section. The analogous hydrolysis reaction and its mechanism have been reported in the literature [24]. The complex is precipitated from the mixture and then purified by thermal gradient sublimation at 290°C, much lower than its decomposition temperature of 356°C (Fig. S1). Single crystals are obtained during sublimation, and the structure is shown with ORTEP and space-filling views in Fig. 1b and c. The complex Ce-2 is a dinuclear compound with two Ce^3+^ ions possessing the same coordination environment (Fig. S2). The center Ce^3+^ ions are well shielded by surrounding ligands (Fig. 1c), which could prevent luminescence quenching. The air stability of Ce-2 powder is quite good—even when exposed to air for 750 hours, the photoluminescence quantum yield (PLQY) of Ce-2 powder does not decrease (Fig. S3).

**Figure 2.**
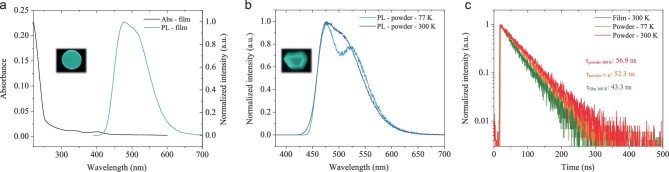
Photophysical properties of Ce-2. (a) Absorption and photoluminescence spectra of Ce-2 neat film. The excitation wavelength is 380 nm. Inset: photograph of Ce-2 neat film on quartz under 365 nm irradiation. (b) Photoluminescence spectra of Ce-2 crystalline powder at 300 K and 77 K; the excitation wavelength is 360 nm. Inset: photograph of Ce-2 crystal under 365 nm irradiation. (c) Transient photoluminescence decays of Ce-2 as neat film and crystalline powder at 300 K and 77 K. The excitation wavelength is 380 nm.

As Ce-2 is insoluble in common solvents, the UV-Vis absorption and photoluminescence spectra are recorded in the thermal evaporated neat film state on a quartz substrate. As shown in Fig. 2a, two absorption bands located at 330 nm and 399 nm with absorbance around 0.01 could be assigned to 4*f*→5*d* transition of Ce^3+^ ions, while strong absorption under 260 nm arises from π–π^*^ transition of the ligand. The Ce-2 neat film exhibits strong emission under UV excitation (Fig. 2a, inset), with a maximum emission peak at 477 nm and a high PLQY of 74%. The crystalline powder of Ce-2 exhibits a similar emission spectrum at room temperature; however, it shows a better resolved emission spectrum with two peaks at 476 nm and 524 nm at 77 K (Fig. 2b). The energy difference between the two peaks is close to 2000 cm^−1^, in agreement with energy splitting between ^2^F_5/2_ and ^2^F_7/2_, the two ground levels of Ce^3+^ ion [20]. The excited state lifetime of Ce-2 neat film is measured as 43.3 ns at room temperature. As for the crystalline powder, 56.9 ns at room temperature and 52.3 ns at 77 K are recorded (Fig. 2c), respectively. All these properties demonstrate that the emission of Ce-2 can be attributed to Ce^3+^ ion, more specifically to the two electric-dipole 5*d*→4*f* transitions of Ce^3+^ ion from the lowest excited state (^2^D_3/2_) to the ground states ^2^F_5/2_ and ^2^F_7/2_.

**Figure 3.**
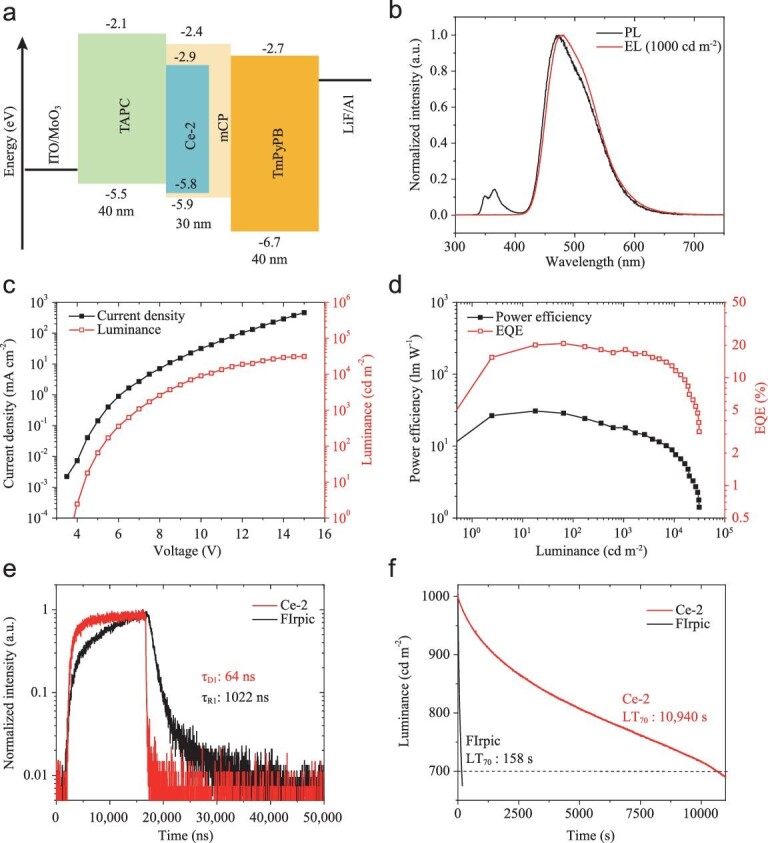
Electroluminescence performance of Ce-2. (a) Schematic device configuration of device D1. The labels give the energy levels in electronvolts and the thickness of layers in nanometers. (b) Electroluminescence spectrum of device D1 and photoluminescence spectrum of the mCP : Ce-2 emission layer. The excitation wavelength is 280 nm. (c) Plot of current density-voltage-luminance characteristics of device D1. (d) Power efficiency-luminance-EQE traces of device D1. (e) Transient electroluminescence decays of device D1 (Ce-2 as the emitter) and R1 (FIrpic as the emitter). (f) Operation lifetime decay of devices D2 (Ce-2 as the emitter) and R2 (FIrpic as the emitter) at an initial luminance of 1000 cd m^−2^.

The complex Ce-2 shows high PLQY and short excited state lifetime, thus is worth investigating as a potential emitter in OLEDs. The electroluminescence properties of Ce-2, including efficiency and operation stability, were studied by fabricating its OLEDs with a vacuum deposition method. The frontier molecular orbital (FMO) energy levels of Ce-2 were estimated by ultraviolet photoelectron spectroscopy (UPS) (Fig. S4) and the absorption edges of the UV-Vis spectrum. Based on the energy levels of Ce-2, 3,3^′^-bis(carbazol-9-yl)biphenyl (mCBP), [9-[3-(9*H*-carbazol-9-yl)phenyl]-9*H*-carbazol-3-yl]diphenylphosphine oxide (mCPPO1), *N,N^′^*-dicarbazolyl-3,5-benzene (mCP) and bis[2-(diphenylphosphino)phenyl]ether oxide (DPEPO) were estimated as host materials during photoluminescence study. The PLQY of mCBP, mCPPO1 and DPEPO with 10% Ce-2 doped films were estimated as 59%, 82% and 75%, respectively. In particular, the mCP doped film exhibits the highest PLQY of 95% and an excited state lifetime of 52.0 ns. Thus it was introduced as the emission layer in OLEDs. Meanwhile, 1-bis[4-[*N,N^′^*-di(4-tolyl)amino]phenyl]cyclohexane (TAPC) and 1,3,5-tri(*m*-pyrid-3-yl-phenyl) benzene (TmPyPB) were used to fabricate hole and electron transport layers, respectively. After optimizing film thickness and doping concentration, the best performance was achieved in device D1 with a structure of ITO/MoO_3_ (2 nm)/TAPC (40 nm)/mCP : Ce-2 (10 wt%, 30 nm)/TmPyPB (40 nm)/LiF (0.7 nm)/Al (100 nm) (Fig. 3a). The device showed no electroluminescence from mCP, which is different from the photoluminescence spectrum of the mCP : Ce-2 (10 wt%) emission layer (Fig. 3b). This is reasonable because the bandgap of mCP is much wider than that of Ce-2, hence carriers may dominantly recombine on dopant rather than host molecules. The device D1 shows a turn-on voltage of 3.9 V, a maximum luminance of 31 160 cd m^−2^, a current efficiency of 45.6 cd A^−1^ and a power efficiency of 30.8 lm W^−1^. The maximum EQE reached 20.8% and remained at 18.2% and 11.6% at 1000 cd m^−2^ and 10 000 cd m^−2^, respectively. This performance is comparable, perhaps even better, than achieved in OLEDs with phosphorescence or TADF materials as emitters [25–28]. For further comparison, a reference device R1 using the classic phosphorescent material FIrpic as the emitter was fabricated, with an identical device configuration. As shown in Table 1, complex Ce-2 shows similar emission color to FIrpic but much higher efficiencies in OLEDs. Notably, the transient electroluminescence lifetimes of devices D1 and R1 are 64 ns and 1022 ns (Fig. 3e), consistent with the excited state lifetimes of the corresponding emitters, Ce-2 and FIrpic, respectively.

**Table 1.** Summarized parameters of key OLEDs in this work.

		V_on_^a^	EQE_max_^b^	EQE_1000_^c^	L_max_^d^	
Device	Emitter	[V]	[%]	[%]	[cd m^−2^]	CIE^e^

D1	Ce-2	3.9	20.8	18.2	31 160	(0.17, 0.33)
R1	FIrpic	3.8	17.4	14.7	31 680	(0.15, 0.31)
D2	Ce-2	3.8	15.3	12.1	102 900	(0.18, 0.35)
R2	FIrpic	3.4	16.2	13.6	18 060	(0.15, 0.33)

^a^Turn on voltage, is taken as a reference point at which the luminance is 1 cd m^−2^. ^b^Maximum EQE. ^c^EQE at 1000 cd m^−2^. ^d^Maximum luminance. ^e^Coordinates at 1000 cd m^−2^.

The electroluminescence stability of Ce-2 is assessed in device D2 with a structure of ITO/MoO_3_ (2 nm)/mCP : MoO_3_ (20 wt%, 30 nm)/mCP (10 nm)/mCP : Ce-2 (10 wt%, 30 nm)/TmPyPB (40 nm)/LiF (0.7 nm)/Al (100 nm) under constant current density at an initial luminance of 1000 cd m^−2^. In D2, we chose mCP as the hole transport material (HTL) rather than TAPC for two reasons: first, TAPC is easily degraded during device operation because of the low bond dissociation energy (BDE) of the C (sp^2^)-N (sp^3^) bond. The higher BDE of the C (sp^2^)-N (sp^2^) bond of mCP leads to better stability [29]; and second, the charge accumulation at interfaces is considered as an important factor in OLED degradation [10]. In D2, the charge barrier between HTL and the emitting layer was eliminated by replacing the TAPC with mCP. Considering the device operation lifetime is greatly affected by materials, device configuration, fabrication environment and encapsulation technique [10], a reference device R2 using FIrpic as the emitter was also fabricated. The performance of these devices is detailed in Table 1 and Fig. S5. Compared to device R2 with an operation lifetime (LT_70_) of 158 s, device D2 showed a dramatically increased LT_70_ to 10 940 s (Fig. 3f). The emission color of device D2 remained stable over a much longer time range, whereas that of device R2 showed substantial change during the aging test (Fig. S5c). Such results indicate that the electroluminescent stability of Ce-2 is significantly better than that of FIrpic. Furthermore, device D2 exhibited much lower efficiency roll-off at high luminance; thus, an ultrahigh maximum luminance over 100 000 cd m^−2^ was achieved. The EQE remained at 11.1% and 8.9% at 10 000 cd m^−2^ and 80 000 cd m^−2^, respectively. The long operation lifetime and small efficiency roll-off can be attributed to the short excited state lifetime of Ce-2.

## CONCLUSION

In summary, we demonstrated a blue emission rare earth cerium(III) complex for high performance OLEDs with a maximum EQE exceeding 20%, and operation stability 70 times greater than that of a typical phosphorescence emitter FIrpic under the same conditions. This excellent performance can be assigned to the almost 100% IQE of the investigated cerium(III) complex and its nanosecond excited state lifetime originating from spin- and parity-allowed 5*d*→4*f* transition of the Ce^3+^ ion. With adjustable emission color and its low cost, the cerium(III) complex could be a new type of emitter for OLEDs.

## METHODS

### Synthesis of Ce-2

KTp^Me2^ (2.68 g, 8 mmol), Ce(CF_3_SO_3_)_3_ (2.34 g, 4 mmol), H_2_O (0.074 g, 4 mmol) and dry tetrahydrofuran (100 mL) were added to a 250 mL round-bottom flask. The mixture was stirred in a glovebox at room temperature for two days. A yellow-green powder was obtained by filtering the suspension, and this was loaded into a thermal sublimator. With gradient temperature of 290°C – 230°C – 130°C and pressure around 2 × 10^−4^ Pa, 0.533 g Ce-2 was obtained as a crystalline powder within 12 hours. Yield: ∼20%. Anal. calcd. for Ce-2: N 21.37%; C 45.82%; H 5.69%; found: N 21.46%; C 45.76%; H 5.65%.

### General characterization

Elemental analyses were performed on a VARIO EL analyzer (GmbH, Hanau, Germany). The crystal structure was obtained with a Rigaku XtaLAB PRO 007HF(Mo) single crystal X-ray diffractometer. UV-vis absorption spectra were recorded on a Shimadzu UV3600Plus UV-VIS-NIR spectrophotometer. Fluorescence and transient PL decay spectra were measured on an Edinburgh Analytical Instruments FLS980 spectrophotometer. PLQYs were measured on a C9920–02 absolute quantum yield measurement system from Hamamatsu Company. Thermogravimetric analysis was undertaken with a Q600SDT instrument. Ultraviolet photoelectron spectroscopy was measured on an AXIS Supra X-ray photoelectron spectrometer.

### OLEDs fabrication and measurement

Indium tin oxide (ITO) patterned anode was commercially available with a sheet resistance of 14 Ω square^−1^ and 80 nm thickness. ITO substrates were cleaned with deionized water, acetone and ethanol. The organic and metal layers were deposited in different vacuum chambers with a base pressure greater than 1 × 10^−4^ Pa. The active area for each device was 4 mm^2^. All electrical testing and optical measurements were performed under ambient conditions with encapsulation of devices in a glovebox. The EL spectra, current density-voltage-luminance (*J−V−L*) and EQE characteristics were measured with a computer-controlled Keithley 2400 source meter and absolute EQE measurement system (C9920–12) with photonic multichannel analyzer (PMA-12, Hamamatsu Photonics).

### Transient electroluminescence measurement

Short-pulse excitation with a pulse width of 15 μs was generated using an Agilent 8114A. The amplitude of the pulse was 9 V, and the baseline was –3 V. The period was 50 μs, delayed time 25 μs and the duty cycle 30%. The decay curves of devices were detected using an Edinburgh FL920P transient spectrometer.

CCDC 1943674 contains the supplementary crystallographic data for this paper. These data can be obtained free of charge from The Cambridge Crystallographic Data Centre via www.ccdc.cam.ac.uk/data_request/cif.

## Supplementary Material

nwaa193_Supplement_File
