# Supplementary material for: Efficient rare earth cerium(III) complex with nanosecond d−f emission for blue organic light-emitting diodes
Source: Natl Sci Rev. 2020 Aug 28;8(2):nwaa193. doi: 10.1093/nsr/nwaa193 (PMC8291366; doi:10.1093/nsr/nwaa193)
Supplement: nwaa193_Supplement_File [file nwaa193_supplement_file.docx]

**SUPPLEMENTARY DATA**

Efficient rare earth cerium(III) complex with nanosecond d-f emission for blue organic light-emitting diodes

Zifeng Zhao, Liding Wang, Ge Zhan, Zhiwei Liu,* Zuqiang Bian, and Chunhui Huang

**Figure S1.** Thermogravimetric analysis of **Ce-2**.


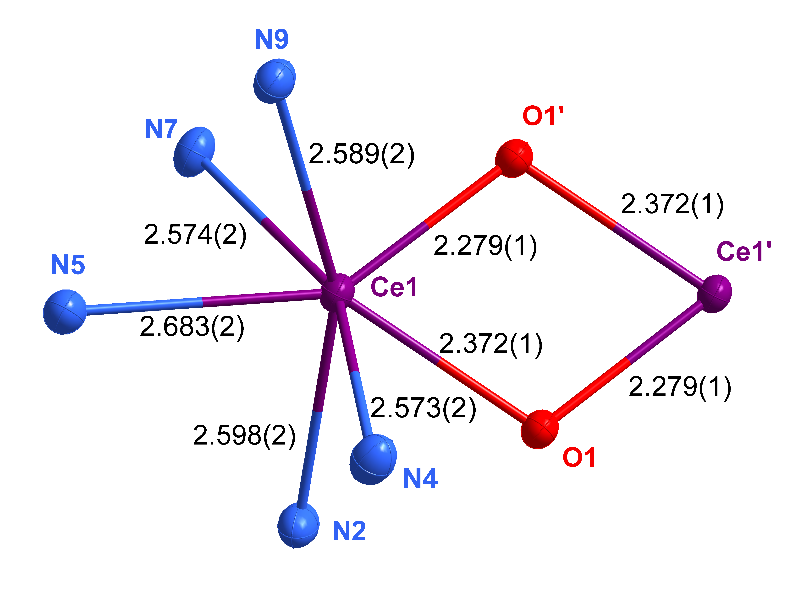


**Figure S2.** The coordination environment of Ce^3+^ ions in **Ce-2**.

**Figure S3.** The PLQY variation of **Ce-2** powder in the ambient environment. Sample storage condition: Temperature, 295 K ~ 303 K. Relative humidity, 30% ~ 60%.

**Figure S4.** Ultraviolet photoelectron spectroscopy of **Ce-2.**

Based on the UPS spectrum and the UV absorption spectrum, the energy of frontier molecular orbitals are calculated as follows:

E_HOMO_ = -(hν-E_B_+E_A_) = -(21.22 eV-18.14 eV+2.73 eV) ≈ -5.8 eV,

E_LUMO_ = E_HOMO_+hc/λ = -5.8 eV+1240/430 eV ≈ -2.9 eV

**Figure S5.** Device characteristics of device **D2** (**Ce-2** as emitter, red line) and **R2** (FIrpic as emitter, black line). **a.** Current density–voltage and luminance–voltage trace. **b.** Power efficiency–luminance and EQE–luminance trace. **c.** CIE coordinates–aging time traces of devices **D2** and **R2**.
